# Three-Dimensional Characterization of Hardened Paste of Hydrated Tricalcium Silicate by Serial Block-Face Scanning Electron Microscopy

**DOI:** 10.3390/ma12121882

**Published:** 2019-06-12

**Authors:** Yongjuan Zhao, Xianping Liu, Bo Chen, Fei Yang, Yongming Zhang, Peiming Wang, Ian Robinson

**Affiliations:** 1 School of Materials Science and Engineering, Tongji University, Shanghai 201804, China; 1730602@tongji.edu.cn (Y.Z.); 1610413@tongji.edu.cn (F.Y.); zym126@tongji.edu.cn (Y.Z.); tjwpm@126.com (P.W.); i.robinson@ucl.ac.uk (I.R.); 2 Key Laboratory of Advanced Civil Engineering Materials (Tongji University), Ministry of Education, Shanghai 201804, China; 3 London Centre for Nanotechnology, University College London, London WC1H 0AH, UK; 4 Division of Condensed Matter Physics and Materials Science, Brookhaven National Laboratory, Upton, NY 11973, USA

**Keywords:** tricalcium silicate (C_3_S), hydration, 3D microstructure, serial block-face scanning electron microscopy (SBFSEM)

## Abstract

With the application of a three-dimensional (3D) characterization technique, serial block-face scanning electron microscopy (SBFSEM), the 3D microstructure of a hydrated cement monomineral, tricalcium silicate (C_3_S), was measured with nanoscale resolution. The 3D morphologies of anhydrous particles, hydrated products, and capillary pores were visualized. Closed and open pores were discovered inside an anhydrous particle. The size and distribution of both the anhydrous C_3_S particles and their capillary pores were analyzed quantitatively and the porosity was determined to be 9%. The distribution of pores was found to be in a good agreement with the inner and outer product model of Hu et. al., with an inner shell distance of 860 nm. Considering the spatial resolution of the instrument and the volume of sample measured, most pores in this experiment could be characterized as capillary pores.

## 1. Introduction

Tricalcium silicate (C_3_S) [1] is the main component in Portland cement, taking up 50–70% of cement mass. The hydration reaction occurs between C_3_S powder and water to form two products, calcium hydroxide (CH) and calcium silicate hydrate (C–S–H) [2,3]. The early-age strength of hardened cement paste can reach about 70% degree of hydration within 28 days, which is attributed to C_3_S [4,5]. For this reason, the hydration of C_3_S has been investigated for several decades [6] and the hydration mechanism is well understood. A variety of techniques have been developed to reveal the influence of material components and microstructure on macro-scale performance and hydration mechanism. Such visualization methods as scanning electron microscopy (SEM) [7,8,9,10,11], transmission electron microscopy (TEM) [12,13,14], atomic force microscopy (AFM) [15,16], and various newly-developed three-dimensional(3D) visualization techniques such as X-ray computed tomography (CT) have played important roles [2,17,18,19,20,21].

Three-dimensional visualization techniques have great advantages in the study of the microstructure of hardened cement pastes, compared with other traditional techniques. For example, while X-ray diffraction (XRD) [7,22,23,24] and thermal analysis [25,26] can be used to analyze the average chemical components and hydration degree of a whole sample, small angle scattering (SAS) techniques, including small angle X-ray scattering (SAXS) and small angle neutron scattering (SANS) can be used to characterize the size of gel particles and gel pores [27,28,29], and X-ray CT can be used to analyze the 3D morphology and distribution of independent micro regions [2,30], which is essential for heterogeneous materials like cement. Compared with 2D techniques such as SEM and TEM, 3D techniques have the advantage of revealing the spatial distribution and the real morphology of irregular-shaped components. In addition, 3D visualization techniques are better than mercury intrusion porosimetry (MIP) in the investigation of pore morphology and pore connectivity [31,32], although MIP is still the traditional way to measure pores in the 1 nm to 1000 μm size range [33].

Like X-ray CT, serial block-face scanning electron microscopy (SBFSEM) is a 3D visualization technique [34]. The concept of SBFSEM was put forward in 1981 [31], and the system of SBFSEM was well-developed by Denk et al. in 2004 [35]. While SBFSEM was developed for use in life sciences, Zankel [36] applied SBFSEM to the study of 3D microstructures of materials in 2009. SBFSEM has widespread potential applications in material science. It has been reported that SBFSEM has been used to image and analyze the 3D microstructures of internal voids, chemical compositions, crack morphology distribution, and interfacial bonding of coatings and adhesives [37,38], alloys [39,40], and zeolite [41]. As an electron microscopy-based 3D technique, SBFSEM can reach about 10–15 nm resolution in three dimensions [37].

The principle of SBFSEM sample preparation is to maintain the microstructure and composition characteristics of samples for the cutting-and-imaging measurement process, since SBFSEM is a destructive method of analysis. Due to the characteristics of the samples, they are usually embedded into epoxy resin and trimmed into a pyramid after hardening to hold the samples (and their structures) during preparation and measurement [42,43].

This current work extends our previous study on C_3_S powders with SBFSEM [44] to the hardened paste of hydrated C_3_S. This expands the application fields of SBFSEM in inorganic, brittle, and heterogeneous materials and demonstrates 3D information on the nanometer scale for the microstructure of hardened cement paste. We observed the 3D morphologies of anhydrous C_3_S, hydrates, and the pore structure of C_3_S after 24 h of hydration. The pore size distributions, porosity, and diameter of the pores have been documented qualitatively.

## 2. Materials and Methods

Monoclinic C_3_S was synthesized by high sintering according to the methods reported by De la Torre et al. [45] and then ground into powder before hydration. The purity of the raw material measured by powder X-ray diffraction using the Rietveld method was about 98%. The synthetic C_3_S was hydrated to a water to C_3_S powder ratio (w/c) of 0.5 at 20 °C for 24 h. Because of the restriction of sample volume by SBFSEM, the hydration of C_3_S was conducted in two steps. Firstly, C_3_S was hydrated in a sealed plastic tube for 5 h. Then, it was removed from the tube and broken into several smaller particles. Then, the particles continued to hydrate at 20 °C with 60% relative humidity for another 19 h.

The hydration of the sample was then stopped by infusion with absolute ethanol. This hydration-stopped sample was then dried and embedded in epoxy resin which was cured at 60 °C for 48 h. The cured sample was trimmed into a frustum-pyramid shape (length of top surface < 500 μm, height < 1 mm) using a Leica microtome (Leica Microsystem Inc., Buffalo., IL., USA) and fixed on the sample holder with superglue for 2 h. Conductive silver adhesive was applied around the surface of the sample in order to increase its conductivity.

Backscattered electron (BSE) images of a series of cross sections of the sample were obtained with a Zeiss Sigma VP scanning electron microscope (Carl Zeiss Microscopy GmbH, Oberkochen, Germany) equipped with a Gatan “3View” in-chamber ultramicrotome (Gatan UK, Abingdon, UK). A Gatan “onpoint” backscattered electron detector (Gatan UK, Abingdon, UK) was used to acquire a series of images from each new section of the sample that was cut by the in-chamber ultra-microtome (Gatan UK, Abingdon, UK). Data were collected in high vacuum mode with a 1.5 kV accelerating voltage and 2 us/pixel dwell time. An image stack with a 50 nm/pixel and 15nm thickness was obtained.

In order to increase the computation speed, original images of 2048 × 2048 pixels were binned into 1024 × 1024 pixels using the ImageJ 2 (Open Source software). Two image stacks obtained from the above procedure were imported separately into Avizo 9.4.0 (Thermo Fisher Scientific Inc, Waltham, MA, USA) for image processing, reconstruction, and analysis. Semi-automatic segmentation and manual segmentation were used in combination to increase the accuracy. Quantitative analysis was also done on the 3D images.

## 3. Results and Discussion

### 3.1. The 3D Microstructure of Hardened Paste of Hydrated C_3_S

**Figure 1:**
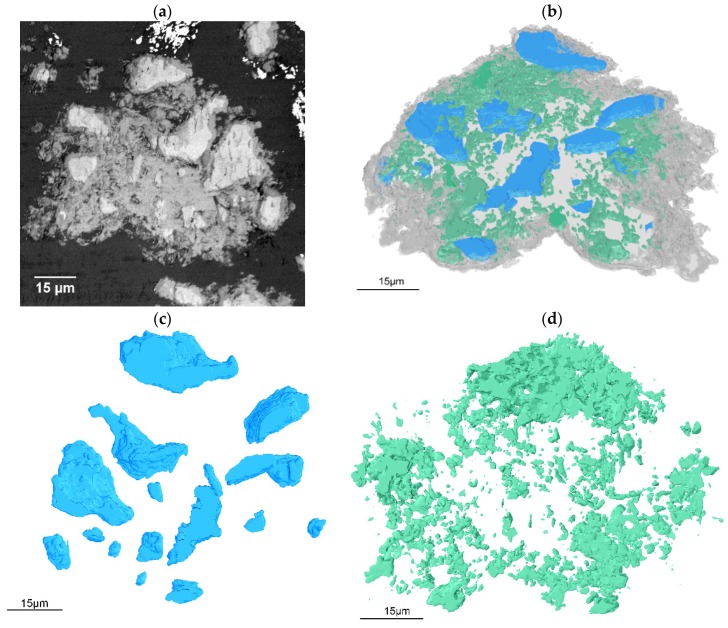
Two-dimensional and three-dimensional images of the hydrated C_3_S: (**a**) the original BSE image of samples, (**b**) the 3D rendered image of the sample, (**c**) anhydrous particles, and (**d**) pores in the hydrated C_3_S paste.

The BSE image of the first section of the hardened paste of C_3_S hydrated for 24 h is shown in Figure 1a. A 3D-rendered image obtained from 380 slices is shown in Figure 1b. According to the atomic number/Composition contrast in the BSE image of Figure 1a, the bright features are anhydrous C_3_S particles, the grey features are hydrates and the dark features are pores and spaces filled by epoxy. These three features are rendered in blue, grey, and green respectively in Figure 1b. Rendered 3D images of anhydrous C_3_S particles and pores, shown in Figure 1c,d, have been qualitatively and quantitatively analyzed to further understand the microstructure characteristics of hardened paste in three dimensions.

### 3.2. Analysis of Anhydrous C_3_S Particles in the Hydrated C_3_S Paste

#### 3.2.1. Morphological Parameters of Anhydrous C_3_S

**Figure 2:**
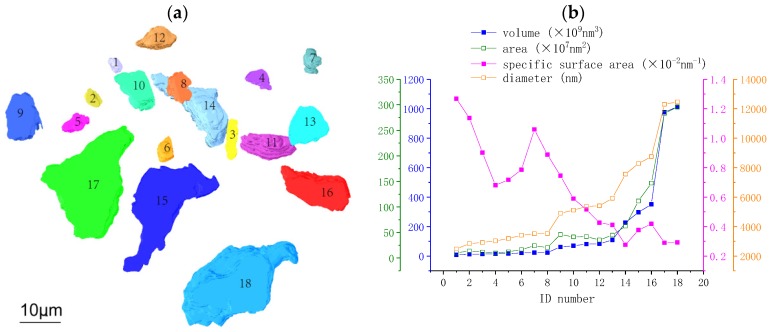
3D image and quantitative analysis of anhydrous particles: (**a**) labeled 3D images of anhydrous particles and (**b**) the volume, area, specific surface area and diameter analysis of anhydrous C_3_S particles.

Figure 2a is a labeled 3D image of the segmented anhydrous C_3_S particles, in which each anhydrous particle is assigned an ID number from 1 to 18. Basic 3D morphological parameters were analyzed using the “label analysis” module in Avizo to provide the volume, the area of the object boundary (labeled as ‘area’), and the specific surface area, as well as the diameter of the spherical particles of the same volume (labeled “diameter”) of all anhydrous particles. The analysis results are shown in Supplementary Spreadsheet S1 and in Figure 2b. The volume ranges from 1.26 × 10^6^ to 1.3 × 10^9^ nm^3^. The diameter of the anhydrous particle ranges from 2500 to 12500 nm, calculated from the equivalent spherical diameter, which is consistent with the volume change. The area changes with the morphology, resulting in the fluctuations in the specific surface area. The general trend is that smaller particles have larger specific surface areas. It is believed that the higher the specific surface area, the higher the hydration rate of anhydrous C_3_S particles due to a larger contact area between water and the particles.

#### 3.2.2. Interface Structure between Anhydrous C_3_S Particles and Hydrates

**Figure 3:**
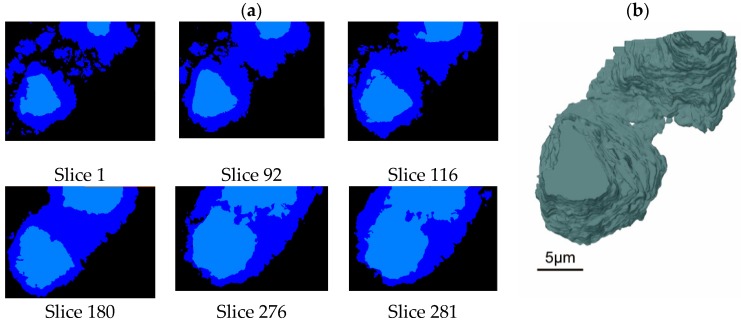
Labeled images and 3D image of hydrated C_3_S: (**a**) labeled images of numbered slices and (**b**) 3D image of an anhydrous particle.

Figure 3a displays the labeled images and a corresponding 3D image of another region of the sample with higher resolution. The lateral (X and Y) pixel size of these data is 20 nm. while the depth (Z) pixel size is 15 nm. The interface between anhydrous C_3_S particles in light blue and hydrates in dark blue is relatively brittle compared with the matrix, but it is of great importance. Although SBFSEM technology is a destructive method, the interface between anhydrous particles and hydrates can be characterized clearly, as shown in Figure 3a where the cutting thickness is 15 nm. The inner hydrate layer surrounding anhydrous C_3_S particles and outer hydrates in the space between anhydrous C_3_S particles can be clearly seen.

As we can see in Figure 3a, two anhydrous particles are separated from each other along with the surrounding hydration products in Slice 1. The hydrated products of the two anhydrous particles start to become connected in Slice 92 and are totally connected in Slice 180. The two anhydrous particles themselves begin to join together in Slice 279 and come to form a whole particle in Slice 281. The 3D image of this region shows that the two anhydrous C_3_S particles presented in Figure 3a, Slice 1, are in fact part of a single curved particle in Figure 3b. It would be easy to come to the wrong conclusion if we had analyzed the structure with just one 2D BSE image like Slice 1. Figure 3 illustrates the advantage of SBFSEM over 2D BSE image technology in the accuracy of characterization of phase morphology. In conclusion, SBFSEM can reveal the 3D characteristics of the morphology, which play an important role in revealing the relationship between the microstructure and performance of materials.

### 3.3. Analysis of Pore Structure in the Hydrated C_3_S Paste

#### 3.3.1. Quantitative Analysis of Pores

**Figure 4:**
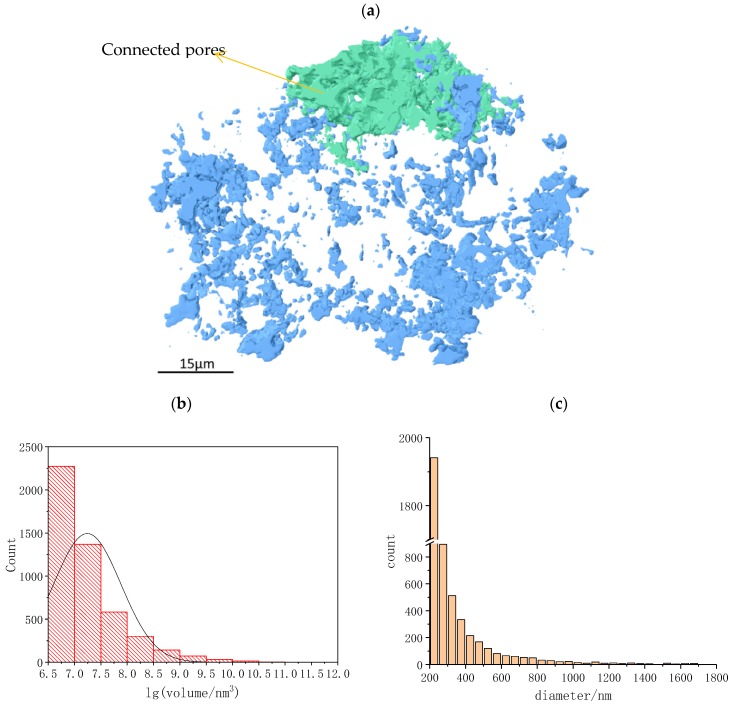
3D image and analysis of pores: (**a**) 3D image of connected pores rendered in green and non-connective pores rendered in blue, (**b**) the volume distribution of all pores, and (**c**) the diameter distribution of all pores.

Pores are one of the most important microstructure characteristics of cement paste. Figure 4a demonstrates the 3D pore network in the hydrates of Figure 1. There are 4800 pores altogether. The large pore cluster rendered in green is a connected pore, which was separated from the other pores through the “axis connective” module (parameter setting: z-axis; pixel > 26). The porosity of connected pores is related to the water transportation ability of the material, which will affect the further hydration of anhydrous C_3_S particles. The porosity of the sample was about 9%, of which connected porosity comprised 4.45%. The pore volume, specific surface area, and pore diameter were analyzed quantitatively with results listed in Supplementary Spreadsheet S2. The volume of pores ranged from 1.2 × 10^6^ to 2.0 × 10^11^ nm^3^. The pore diameter ranged from 200 to 15000 nm, and 99% of pore diameters were below 2000 nm.

The lognormal distribution of pore volume and the frequency distribution of pore diameter (<2000 nm) are listed in Supplementary Spreadsheet S2 and shown in Figure 4b,c. The average pore volume was 1.7 × 10^7^ nm^3^. The 95% confidence interval of the volume logarithmic distribution was (7.224, 7.260) which means 95% particles are between 1.6 × 10^7^ and 1.8 × 10^7^ nm^3^. The average diameter of pores was 380 nm. According to Ma et al.’s study [10], pores in concrete can be classified into gel pores ranging from 0.5 to 10 nm, capillary pores ranging from 10 nm to 10 um, and macropores above 10 um. As shown in Figure 4b, nearly all pores in the sample were capillary pores. No macropores were detected. The size of the gel pore is lower than the spatial resolution of SBFSEM and therefore cannot be identified.

The assessment of pores in cement paste has been studied for several decades. Mercury intrusion porosimetry (MIP) is the widely accepted method to measure open pores ranging from 1 nm to 1000 μm. Although the pore size range that SBFSEM can detect in our study is smaller than that of MIP, it still has great advantage in the study of the capillary pore structure of cement-based materials. Compared with MIP, SBFSEM can obtain not only the size distribution but also morphology and connectivity parameters of both open pores and closed pores. Since capillary pores play an important role in the strength, permeability, and shrinkage properties of hardened cement paste, accurate characterization of them is of great practical significance. From the MIP results of former literature, the porosities of typical cement materials are between 10% and 40% [46], but the porosity of our sample is about 9%. Although the porosity seen by SBFSEM is in the range of 10–40%, this apparent agreement is coincidental in consideration of the different pore types that the two methods can detect. While MIP can detect gel pores, capillary pores, and macropores, SBFSEM can detect both closed and open pores. Based on the imaging quality of SBFSEM in our study, influenced by the weak conductivity of samples and the cutting thickness along the z-axis, the spatial resolution of the images in three dimensions is around 50 nm. 

#### 3.3.2. The Spatial Distribution of Pores

**Figure 5:**
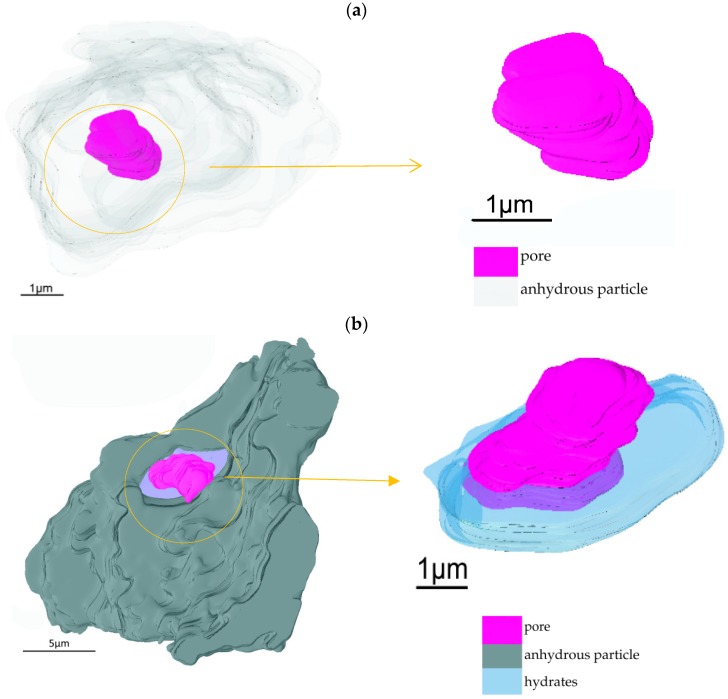
Two types pores in the anhydrous C_3_S particles: (**a**) closed pores and (**b**) open pores.

Additional pores were found to be located in the anhydrous particle of No. 17 (labeled in Figure 2a). These are believed to occur by sublimation during the solid phase sintering and are displayed in Figure 5. Figure 5a presents the closed pore, while in Figure 5b, the anhydrous particles, hydrates and open pores in the anhydrous particle are displayed separately in transparent gray, blue, and purple colors. The pore located next to the boundary of the anhydrous particle is mostly surrounded by hydrates, and hydrates can also be viewed within a certain thickness of the inner wall of the pore. This arrangement suggests that the pore is more like an open pore than a closed pore. 

Open pores have an important relationship with water transportation and hence with hydration. Given that hydrates lie at the inner wall of the pore rather than filling the whole pore, this suggests that the open pore may have originated as closed pore (filled with air) in the anhydrous C_3_S particle. During hydration, this closed pore opened and the hydration reaction started at the interface between pores and hydrates and then gradually deposited around the inside of the pore.

**Figure 6:**
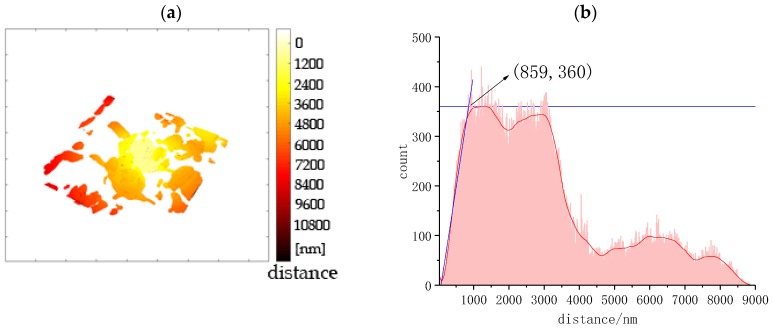
Distance of capillary pores from the surface of the anhydrous C_3_S particle qualitatively (**a**) and quantitatively displayed (**b**).

The distance from the capillary pores to the surface of the anhydrous particle was computed through the “surface distance” module in the Avizo software, which calculates the distance from each voxel on surface 1 to each voxel on surface 2. To assist the distance computation, a small anhydrous particle, No. 2 in Figure 2a, was chosen and then the image was cropped into a 15 × 14 × 5.7 μm region. Results are displayed in Figure 6 and Table 1, as well as in Supplementary Spreadsheet S3. In Figure 6 the pore network around the anhydrous particles is visualized using the shade of color to represent the distance from the anhydrous particle. The shortest distance between the pores and the anhydrous particles is 30 nm, and the mean value is 4400 nm (Table 1). Distances below 860 nm make up only 0.4% of all pixels which means that almost all capillary pores are located more than 860 nm away from the anhydrous particle. According to Hu et al.’s study [2], the hydration products can be divided into inner hydration products (IP) with high density and outer hydration products (OP) with low density. Figure 1a clearly shows the difference in pore structure between IP and OP in the BSE image. Capillary pores tend to be found in the OP rather than IP. The demarcation between the IP and OP of the anhydrous particle can be better identified using the spatial distribution of capillary pores [47], even though they are difficult to distinguish through gray levels. From the above analysis, the thickness of IP is 860 nm, and the OP starts from 860 nm and is further away. This analysis method can be used to identify the IP and OP using SBFSEM quantitatively. This new information will be of benefit to the research on 3D microstructure evolution during cement hydration and hence improve our knowledge of the hydration mechanism.

## 4. Conclusions

A recently-developed 3D electron microscopy technique, SBFSEM, was successfully applied to characterize the 3D microstructure of a cement monomineral, C_3_S, which is a brittle, hard, and porous inorganic material, after hydration for 24 h. Due to the poor conductivity of inorganic material, the spatial resolution in this study was about 50 nm which made it possible to observe the 3D spatial structure of materials at the nanoscale. The morphology, size and distribution of anhydrous particles, hydrates, and pores were analyzed quantitatively. The volume of anhydrous C_3_S particles ranged from 1.26 × 10^6^ to 1.3 × 10^9^ nm^3^. The diameter of the anhydrous particles ranged from 2500 to 12500 nm. The above results are important for the study of hydration kinetics of C_3_S particles. The volume of pores ranged from 1.6 × 10^7^ to 1.8 × 10^7^nm^3^ and their diameters ranged from 200 to 15000 nm. The average pore volume and diameter were 1.7 × 10^7^ nm^3^ and 380 nm. The locations of open and closed pores associated with hydrates were found 860 nm away from the anhydrous particles. SBFSEM can be further applied to characterize the evolution of 3D microstructure of hardened cement paste at nanoscale. The 3D analytical results will help us to understand the cement hydration mechanism.

## Figures and Tables

**Table 1:** The critical distance from pores to anhydrous particles.

	Min	Mean	Max	Inflexion
Distance/nm	30	4400	9000	860

## Supplementary Materials

The following supplementary materials are available online at https://www.mdpi.com/1996-1944/12/12/1882/s1, Spreadsheet S1: volume, area, specific surface area, and diameter of 18 anhydrous C_3_S particles in Figure 2a; Spreadsheet S2: volume, specific surface area, and diameter of pores below 2000 nm in Figure 4a; Spreadsheet S3: distance of capillary pores from the surface of the anhydrous C_3_S particle in Figure 6a.
